# Protein Post-Translational Modifications in Plant Abiotic Stress Responses

**DOI:** 10.3390/plants15010052

**Published:** 2025-12-23

**Authors:** Gengmi Li, Baohua Feng, Qian-Hao Zhu, Kaifeng Jiang, Tao Zhang

**Affiliations:** 1 Key Laboratory of Southwest Rice Biology and Genetic Breeding, Ministry of Agriculture/Luzhou Branch of National Rice Improvement Center, Rice and Sorghum Research Institute, Sichuan Academy of Agricultural Sciences, Deyang 618000, China; wilfred123@163.com; 2 State Key Laboratory of Rice Biology and Breeding, China National Center for Rice Improvement, China National Rice Research Institute, Hangzhou 310006, China; fengbaohua@caas.cn; 3 Commonwealth Scientific and Industrial Research Organization, Agriculture and Food, GPO Box 1700, Canberra, ACT 2601, Australia; qianhao.zhu@csiro.au

**Keywords:** phosphorylation, ubiquitination, SUMOylation, PTMs, abiotic stress response

## Abstract

Protein post-translational modifications (PTMs), as an important biological process of plants responding to environmental stimuli, can regulate the chemical decoration and properties of translated proteins by altering amino acid side chains or protein terminal structures, thereby affecting the synthesis, assembly, localization, function, and degradation of proteins. Notably, PTMs regulate protein function without changing protein expression levels. Two dozen types of PTMs have been identified. This review summarizes the molecular mechanisms of major types of PTMs, including phosphorylation, ubiquitination, SUMOylation, glycosylation, methylation, and acetylation, with a focus on their regulatory roles in plant responses to abiotic stresses. Under heat stress, phosphorylation activates transcription factors such as HSFA1 (heat shock transcription factor 1), while SUMOylation regulates the activity of HSFA1/HSFA2 in the heat stress signaling pathway. Upon cold stress, phosphorylation, ubiquitination, and S-acylation collectively regulate the expression of cold tolerance-related genes. The drought stress response relies on SnRK2s (Sucrose 321 non-Fermenting 1-related protein kinase 2s) -mediated phosphorylation, regulation of ARF7 (auxin response factor 7) by SUMOylation, and ubiquitination. In salt stress, the coupling of phosphorylation of SOS (salt overly sensitive) pathway-related proteins, ubiquitination, and phospholipid metabolism maintains ion homeostasis. Additionally, PTMs play a key role in ABA-mediated abiotic stress responses by regulating core components of signal transduction, such as PYR (pyrabactin resistance)/PYL (PYR1-LIKE)/RCAR (regulatory components of ABA receptor) receptors, PP2Cs (protein phosphatases type 2C), and SnRK2s. On the basis of the synthesis of the regulatory mechanisms of PTMs, we discuss how PTMs can be manipulated to breed abiotic stress resilient crops and the issues to be addressed to achieve the goal, such as crosstalk between PTMs, technical challenges in investigating PTMs and identifying PTM substrates.

## 1. Introduction

Plants are constantly exposed to a variety of unsuitable environments, limiting their growth and development, particularly under the reality of global warming. Protein post-translational modification (PTM) is the process of chemically modifying proteins after they are translated. Many types of PTMs have been documented. The Plant PTM Viewer 2.0 (https://www.psb.ugent.be/webtools/ptm-viewer/ptm_info.php (accessed on 11 November 2025)) lists 24 types of PTMs covering 437,404 PTM sites of 143,688 proteins. Some of these PTMs occur at specific amino acid residue(s), some at the C- or N-terminus of proteins. These modifications impact stability and localization of proteins, and assembly of protein complexes, thereby regulating functions of proteins without altering their expression level [1]. PTMs play a pivotal role in plants’ responses to environmental stimuli, occurring critically and rapidly in response to changing environmental conditions, so having the potential to be harnessed for improving plants’ capacity to combat the effects of adverse environments.

This review summarizes the research progress of PTMs in plants. Given the scope (both the diverse types of PTMs and the number of plant species) of the studies on plant PTMs, our focus here was on the main types of PTMs, including phosphorylation, ubiquitination, SUMOylation, glycosylation, methylation, and acetylation investigated in model monocotyledonous (rice) and dicotyledonous (*Arabidopsis*) species, and their functions in abiotic stress response. On the basis of synthesizing the main findings, we propose strategies by which PTMs could be employed in breeding climate-smart and abiotic stress resilient crops.

## 2. Main Types of Post-Translational Modifications

### 2.1. Phosphorylation

Protein phosphorylation is catalyzed by protein kinases and has the most diverse target proteins (Table 1). Protein kinases mediate the reaction by transferring the γ-phosphate group—the terminal, high-energy phosphate moiety—from nucleotide triphosphates (typically adenosine triphosphate (ATP), and occasionally guanosine triphosphate (GTP)) to specific amino acid residues of target proteins [2]. It was first identified by Levene and Alsberg in egg yolk protein [3]. In *Arabidopsis thaliana*, around 41% of the expressed proteins are phosphorylated by protein kinases and the phosphorylation sites are mainly serine (Ser), threonine (Thr), and tyrosine (Tyr) residues and rarely on N-terminal under different conditions [4,5]. Recently, the combination of TurboID-based proximity labeling (TbPL) with mass spectrometry (hereafter referred to as TbPL-MS) and the multiplexed assay for kinase specificity (MAKS) has significantly accelerated research advancements in the field of protein phosphorylation [6,7,8], leading to the identification of more than 43,000 phosphorylation sites in *Arabidopsis* [5]. Phosphorylation changes the features of proteins involved in mitogen-activated protein kinases (MAPKs), calcium-dependent protein kinases (CDPKs/CPKs), calcineurin-interacting protein kinases (CIPKs), and so on. All of them play pivotal roles in the abiotic stress response [9,10].

### 2.2. Ubiquitination

**Figure 1:**
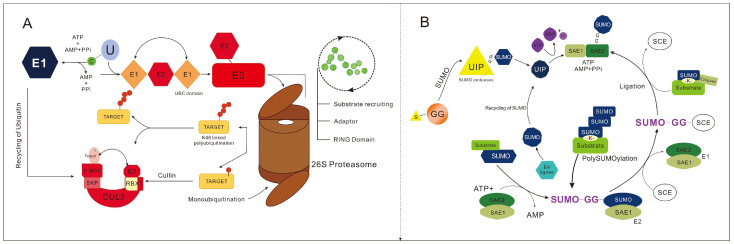
Ubiquitination and SUMOylation in plants. (**A**) Ubiquitination regulation pathway; (**B**) SUMOylation regulation pathway.

Ubiquitin is a small-molecule protein that is widely present in eukaryotes and highly conserved. It contains 76 amino acids with a molecular weight of approximately 8.5 kD. The ubiquitination process is catalyzed by a cascade of three enzymes, namely ubiquitin-activating enzyme E1, ubiquitin-conjugating enzyme E2, and ubiquitin-ligase enzyme E3 [11]. The ubiquitination process is reversible: deubiquitinating enzymes (DUBs) can hydrolyze the ubiquitin chains on target proteins, recycle ubiquitin molecules, and thus maintain the homeostasis of the ubiquitin pool [12]. Ubiquitin-activating enzyme E1 utilizes energy provided by ATP to activate ubiquitin (Ub) molecules, forming a Ub-E1 complex. Through transesterification, the Ub-E1 complex transfers Ub molecules to ubiquitin-conjugating enzyme E2, resulting in the formation of an Ub-E2 complex. Finally, under the action of ubiquitin-ligase enzyme E3, Ub molecules are covalently linked to the lysine (Lys) residues of the target protein(Figure 1A) [13,14]. Ubiquitination modification participates in a variety of biological processes by influencing protein stability, protein subcellular localization, and protein–protein interactions, to regulate cell cycle, circadian rhythm, hormone signal transduction, growth and development, as well as responses to abiotic and biotic stresses [15]. The rice genome contains 6 genes encoding E1 ubiquitin-activating enzymes, 48 genes encoding E2 ubiquitin-conjugating enzymes or E2-like proteins, more than 1500 genes encoding E3 ubiquitin-ligases, and 95 genes encoding deubiquitinating enzymes (DUBs); these enzymes target key components involved in numerous biological processes in rice, such as plant growth and responses to abiotic and biotic stresses [16,17].

### 2.3. SUMOylation

A Small Ubiquitin-like Modifier (SUMO) molecule consists of approximately 100 amino acids. It shares only 12–16% sequence similarity with ubiquitin, yet the two proteins form highly similar three-dimensional structures after folding. Like ubiquitination modification, SUMOylation is also an ATP-dependent cascade enzymatic reaction: under the combined action of specific E1 activating enzymes, E2 conjugating enzymes, and E3 ligases, mature SUMO molecules covalently bind to the Lys residues of target proteins via isopeptide bonds (Figure 1B) [18]. SUMOylation often regulates protein stability, activity, structure, localization, and protein–protein interactions by competing with ubiquitination modification sites, and is also involved in various biological processes such as transcriptional regulation, cell cycle regulation, and environmental response [19,20].

### 2.4. Glycosylation

The glycosylation modification of proteins mainly occurs in the Golgi apparatus and the endoplasmic reticulum. Under the action of various glycosyltransferases, sugar chains bind to amino acid residues on polypeptide chains and form glycosidic bonds [21]. The newly generated glycosylated proteins undergo further transport, splicing, and modification, and finally exert their functions at specific sites. There are four main types: N-glycosylation, O-glycosylation, C-mannosylation, and glycosylphosphatidylinositol (GPI)-mediated glycosylation, among which the first two are the most prominent types [22,23,24]. N-glycosylation modification refers to the covalent attachment of N-glycans to asparagine residues in the consensus sequence Asn-X-Ser/Thr/Cys (where X represents any amino acid except proline) of polypeptide chains via N-glycosidic bonds [25,26]. In contrast, O-glycosylation modification occurs through the formation of O-glycosidic bonds between the hydroxyl termini of serine or threonine residues in the protein peptide chain and glycosyl groups [27].

### 2.5. Methylations

Under the catalysis of different protein methyltransferases, methyl groups can be transferred from the methyl donor S-adenosylmethionine (SAM) to the side chains of amino acids such as arginine and lysine. Most protein methylation processes are dynamic and reversible, with the dynamic balance co-regulated by methyltransferases and demethylases [28]. Protein methylation exhibits amino acid preference, predominantly occurring on arginine and lysine residues. Among these, arginine methylation is catalyzed by protein arginine methyltransferases (PRMTs) [29]. To date, nine PRMTs have been identified [30]. They catalyze arginine monomethylation, symmetric dimethylarginine (SDMA), or asymmetric dimethylarginine (ADMA) [31]. In contrast to arginine methylation, lysine can undergo monomethylation, dimethylation, and trimethylation [32]. Lysine methylation is catalyzed by protein lysine methyltransferases (PKMTs) and demethylated by lysine demethylases (KDMs).

### 2.6. Acetylation

Lysine acetyltransferases catalyze the transfer of acetyl groups to the ε-amino side chains of lysine residues, forming protein acetylation modifications, which can be reversed by lysine deacetylases. In plant mitochondria, some lysine acetylation events can also occur through non-enzymatic reactions [33]. Lysine acetylation includes both histone and non-histone acetylation. Histone lysine acetylation mainly occurs at the N-terminus of core histones and is a key post-translational modification that influences chromatin structure. Lysine acetylation facilitates the dissociation of histones from DNA octamers and induces nucleosome relaxation, thereby enabling specific binding of various transcription factors to DNA binding sites and regulating gene expression levels [34]. Numerous studies have demonstrated that reversible and rapid changes in histone acetylation play crucial regulatory roles in plant growth and development, as well as abiotic/biotic stress responses [34,35,36]. Non-histone acetylation is relatively conserved evolutionarily from bacteria to mammals. Non-histone acetylation regulates various biological processes by modulating enzyme activity [37,38], protein–protein interactions, and protein stability [39,40]. Most molecular functions of plant non-histone acetylated proteins are associated with catalytic activity and binding, and their roles in plant seed germination and reproductive growth have been extensively investigated [41,42,43].

### 2.7. S-Nitrosylation

As an important signaling molecule, nitric oxide (NO) is extensively involved in regulating various physiological and biological processes across diverse organisms. S-nitrosylation is a NO-dependent and reversible redox-based post-translational modification (PTM), in which the NO group is covalently attached to the free sulfhydryl group of a specific cysteine residue in the target protein, thereby forming S-nitrosothiol (SNO) [44]. S-nitrosylation is widely present in various organisms and regulates multiple biological processes in plants, such as growth, immune response, stress response, chlorophyll metabolism, and photosynthesis [45,46]. It has become the primary mechanism for transmitting the biological activity of NO in abiotic stress responses [47]. Its regulatory functions are primarily manifested in five aspects: Firstly, S-nitrosylation can affect protein stability by altering protein half-life to modulate protein abundance; secondly, this modification can directly regulate the biochemical activities of proteins, including enzyme activity and catalytic efficiency; thirdly, S-nitrosylation can induce conformational changes in proteins, thereby altering their functional states; fourth, it can regulate the subcellular localization of proteins, influencing their spatial distribution within cells; finally, S-nitrosylation can modulate protein–protein interactions, which in turn affects signal transduction pathways and molecular networks [48,49,50].

### 2.8. Other PTMs

Lysine crotonylation is an evolutionarily conserved acylation modification. It mainly occurs on lysine residues of histones, and is primarily distributed on histones in the promoter regions or potential enhancer regions of active genes. This modification is widely involved in regulating the initiation, activation, and expression of genes, thereby affecting the physiological processes of plants [51]. In rice seedlings, 1265 lysine crotonylation sites have been identified on 690 proteins. Among these, 51% of the lysine-crotonylated proteins are located in chloroplasts, and the degree of lysine crotonylation modification is positively correlated with the gene expression levels [52].

**Table 1:** Representative major types of PTMs and their functions in plants.

Types of PTM	Modified Amino Acid Residue	Number of Proteins with Modifications	Main Functions	Ref.
Phosphorylation	S, T, Y, D, N-term	82,207	Plant growth and development, response to abiotic/biotic stresses, substance metabolism, energy balance, intracellular homeostasis, and signal network integration	[53]
Ubiquitination	K, N-term	8315	Timing and homeostasis of plant growth and development, response to abiotic/biotic stresses, substance metabolism, energy balance, intracellular homeostasis, and signal network integration	[54]
SUMOylation	K	139	Response to abiotic/biotic stresses, timing and homeostasis of plant growth and development, maintain genome stability, substance metabolism, energy balance, intracellular homeostasis, and signal network integration	[55]
N-glycosylation	N, P, R	3258	Protein folding, plant growth and development, response to biotic stresses, substance metabolism, energy balance, intracellular homeostasis, and signal network integration	[56]
Methylation	R, K, C	1299	Epigenetic regulation	[57]
Acetylation	K, N-terminus	23,042	Epigenetic regulation	[34]
S-nitrosylation	C	5924	Response to abiotic/biotic stresses, plant growth and development	
Crotonylation	K	15,404	Photosynthesis, various substance metabolisms, and protein synthesis and quality control	[58]
2-Hydroxyisobuturylation	K	34,884	Gene transcription, balance of substance metabolism, and response to biotic stresses	[59]
S-sulfenylation	C	3871	Cellular redox homeostasis	[60]
Reversible Cysteine Oxidation	C	3826	Cellular redox homeostasis	[61]
Succinylation	K	2450	Substance metabolism, energy balance, cellular homeostasis, and epigenetic regulation	[59]
S-Acylation	C, N-term	1524	Plant growth and development	[60]
Carbonylation	R, K, P, T, Y	188	Clearance of damaged proteins, and maintenance of cellular homeostasis	[62]
S-cyanylation	C	132	Cyanide metabolism and toxicity detoxification	[60]

## 3. Post-Translational Modifications in Plant Abiotic Stress

### 3.1. Heat Stress

**Figure 2:**
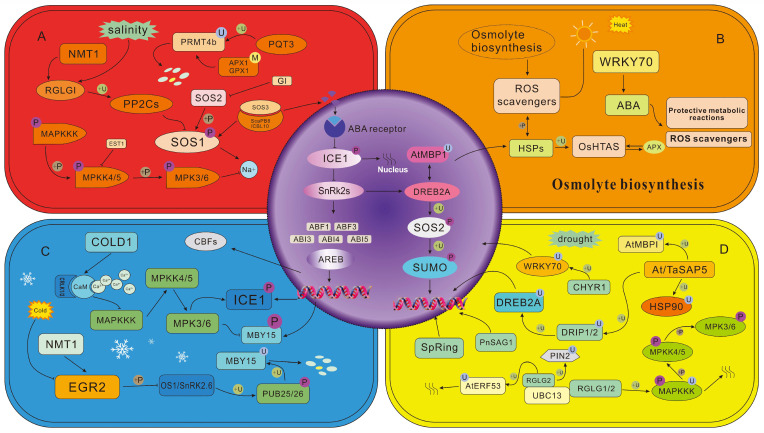
Post-translational modifications in plant abiotic stress. (**A**) Salt stress. (**B**) Heat stress. (**C**) Cold Stress. (**D**) Drought Stress.

Typically, a temperature that is 5~10 °C higher than the optimal growth temperature for plants is sufficient to induce the heat stress response. Some studies suggest that the perception site for high temperatures is the plasma membrane [63]. The Cyclic Nucleotide-Gated Ca^2+^ Channels (CNGCs) and ANNEXIN1 (ANN1) in the plasma membrane respond to the increase in membrane fluidity caused by high temperatures, promoting the entry of apoplastic Ca^2+^ into the cytoplasm. Under high-temperature conditions, the content of cytoplasmic cAMP increases and can activate CNGCs. Researchers believe that enzymes with adenylate cyclase activity are also among the membrane-associated temperature receptors. Ca^2+^ signals are transduced by calmodulin (CaM) and calcium-dependent protein kinases (CDPKs). During this process, Ca^2+^ initiates a specific signal transduction cascade that can activate kinases by binding to CaM associated with CNGCs. The activated kinases then phosphorylate and activate HSFA1, leading to the dissociation of HSP70/HSP90 that interacts with HSFA1 (Figure 2B). This sequence of events ultimately activates the signal transduction pathway in plants in response to heat stress [64,65,66].

The transcriptional regulatory network under heat stress requires the involvement of multiple transcriptional regulators. Heat shock transcription factor (HSF) is a key regulator of heat shock response transcription; the binding activity of yeast HSF to target sites differs by more than 200-fold between 15~39 °C. Although HSF in all states can bind to DNA, its activity is affected by its phosphorylation status [67]. In plants, HSFA1 (heat shock transcription factor 1), HSFA2, and HSFA3 are all important transcription factors involved in response to heat stress [68,69,70]. When plants are subjected to heat shock stress, they rapidly adjust the expression patterns of related genes to adapt to the elevated ambient temperature. Among these regulators, HSFA1s act as core transcription factors in the plant heat shock response, helping to regulate the expression of numerous downstream heat shock-responsive genes [71].

The activity of HSFA1 is tightly regulated at the protein level, primarily through PTMs and protein–protein interactions. In *Arabidopsis thaliana*, protein kinases that interact with HSFA1 include CDC2a (cyclin-dependent kinase 2a), CBK3 (CaM-binding protein kinase 3), and PP7 (Calmodulin-binding Protein Phosphatase 7). These kinases can regulate the heat shock response process by phosphorylating or dephosphorylating HSFA1 [72,73,74]. HSFA1 may also undergo SUMOylation during the heat shock response, and this modification inhibits the activity of HSFA1 [75]. Additionally, under normal temperature conditions, HSFA1 can form a protein complex with HSP70, thereby inhibiting HSFA1 activity. When cells are subjected to heat shock stress, the concentration of misfolded proteins in the cell increases. These denatured proteins competitively bind to HSP70 with HSFA1, thereby releasing HSFA1 and activating the expression of downstream heat shock-responsive genes [76,77].

SUMOylation participates in plant acquired thermotolerance by regulating HSFA2. Specifically, SUMO1 can bind to the lysine at position 315 of HSFA2, thereby inhibiting its transcriptional activity. In plants overexpressing *SUMO1*, the expression of downstream HSPs is significantly reduced after heat treatment [78]. In contrast, SUMO1 can also specifically modify the lysine at position 298 of HSFA1, which promotes the latter’s transcriptional activity [79]. DREB2A (dehydration-responsive element binding protein 2A) is also a core transcription factor involved in the response to high-temperature stress. Under normal conditions, DREB2A is unstable and degraded via the 26S proteasome pathway. However, under high-temperature conditions, SIZ1-mediated SUMOylation of DREB2A inhibits the interaction between DREB2A and the proteasomal degradation system, thereby enhancing the stability of DREB2A protein [80]. DREB2A is known to upregulate *HSFA3* expression, implying that DREB2C might also be implicated in the regulatory network of HSFA3 [81].

The activity regulation of HSFA1 is a typical case of crosstalk between phosphorylation and SUMOylation. Phosphorylation by kinases such as CDC2a activates HSFA1, while SUMOylation at the K298 residue further enhances its transcriptional activity; in contrast, SUMOylation of HSFA2 at the K315 residue represses its function. This residue-specific crosstalk pattern suggests that SUMOylation acts as an elaborate molecular switch governing the HSF family. However, dynamic tracking data are currently lacking to clarify whether phosphorylation affects the residue selectivity of SUMOylation and the temporal regulatory relationship between these two modifications (i.e., whether phosphorylation precedes SUMOylation). In addition, the crosstalk synergistic effects among different HSF members (e.g., whether phosphorylation of HSFA1 facilitates SUMOylation of HSFA2) have not been thoroughly investigated; most existing studies focus on individual members, lacking an integrated analysis of the global modification network of the family proteins.

### 3.2. Cold Stress

Under cold stress, the first rapid response factors are these key cold sensors, including COLD1/RGA1, Ca^2+^ channels, and plasma membrane-localized receptor-like kinases (RLKs) [82]. Among them, COLD1/RGA1 and Ca^2+^ channels have been the subject of extensive research. *COLD1* encodes a G-protein regulator, which is localized to the plasma membrane and endoplasmic reticulum. It is the first cold sensor identified in rice. When plants are exposed to cold stress, COLD1 interacts with the G-protein α subunit RGA1 to activate Ca^2+^ channels, thereby promoting Ca^2+^ influx and triggering the signal pathway for low-temperature response [83]. After perceiving low temperatures, plants rapidly respond to cold stress through the MAPK cascade signal transmission and the ICE1-CBF-COR module [84]. CNGCs, Glutamate Receptor-Like Channels (GLRs), and Mid1-Complementing Activity 1/2 (MCA1/MCA2) channels with complementary activities are the main Ca^2+^ channel proteins involved in cold signal perception [85]. CNGCs are a class of non-selective cation channels localized to the plasma membrane. They activate the cAMP or cGMP cyclase activity by binding to G-protein-coupled receptors, leading to the release of cAMP or cGMP and the opening of Ca^2+^ channels [86]. In rice, the cyclic nucleotide-gated channels OsCNGC14, OsCNGC16, and OsCNGC9 all positively regulate the process of rice resistance to cold stress. After mutation of the OsCNGC14 and OsCNGC16 genes, cold-induced Ca^2+^ influx decreases, and the plants become sensitive to cold stress [87]. The opening and closing of the Ca^2+^ channel controlled by OsCNGC9 is regulated by the phosphorylation of the OsSAPK8 kinase. Under cold stress conditions, OsCNGC9 is phosphorylated, the Ca^2+^ channel opens, and the cold tolerance of rice plants is enhanced [88]. MCA1 and MCA2 are two calcium-permeable mechanosensitive channel proteins in *Arabidopsis thaliana*. They convert extracellular mechanical signals (including stretch, pressure, gravity, and osmotic pressure) into intracellular Ca^2+^ fluxes to transmit cold signals [89].

Plant kinase cascade reactions are crucial processes in the plant’s cold stress response, linking cold signals to cellular responses through MAPK cascade reactions [90]. After MAPKK (Mitogen-Activated Protein Kinase Kinase) and two calmodulin-regulated receptor-like kinases, CRLK1 (Calmodulin-Regulated Receptor-Like Kinase 1) and CRLK2, receive cold signals, they transmit the signals via the MEKK1-MEK2-MPK4 kinase pathway. Furthermore, the MEKK1-MEK2-MPK4 pathway inhibits the phosphorylation of ICE1 (Inducer of CBF Expression 1) by MPK3/6 through antagonizing the MKK4/5-MPK3/6 reaction, thereby enhancing the plant’s ability to resist cold stress [91]. In addition, under cold stress, CRPK1 (Cold-Responsive Protein Kinase 1) can phosphorylate 14-3-3 proteins. The phosphorylated 14-3-3 proteins, acting as shuttle proteins, translocate from the cytoplasm to the nucleus, where they bind to the nucleus-localized CBF1/3 (C-repeat Binding Factor 1/3) and promote their degradation, thus negatively regulating the plant’s cold tolerance process [92].

ICE1 is the primary regulator of CBFs (C-Repeat Binding Factors) and a member of the bHLH (basic Helix–Loop–Helix) transcription factor family. It positively regulates plant cold tolerance through the ICE1-CBF-COR pathway. ICE1 can bind to the promoter region of CBF genes to regulate CBF expression, and CBFs, acting as transcription factors, activate the expression of *COR* (Cold-Responsive) genes—key genes involved in the plant’s defense against cold stress. In *Arabidopsis thaliana*, MPK3/MPK6 (Mitogen-Activated Protein Kinase 3/6) phosphorylates ICE1, leading to a decrease in ICE1 stability and thereby negatively regulating plant cold tolerance [84]. In contrast, phosphorylation of ICE1 by OsMPK3 increases ICE1 stability, enhancing the plant cold stress tolerance in rice [93]. These results indicate that the core molecular modules for cold signal perception and transmission exhibit functional divergence across different plant species. *Arabidopsis* OST1 (Open Stomata 1), also known as SnRK2.6 (Sucrose Non-Fermenting 1-Related Protein Kinase 2.6), is a key regulator in cold signal transduction. Under cold stress, OST1 can bind to and phosphorylate ICE1, preventing ICE1 from being degraded by the 26S proteasome. This process enhances the transcriptional activity of CBFs and improves plant cold tolerance [94,95]. *HOS1* (High Expression of Osmotically Responsive Gene 1) encodes an E3 ubiquitin ligase. It negatively regulates plant cold tolerance by ubiquitinating ICE1. The protein kinase BIN2 (Brassinosteroid-Insensitive 2) further modulates the plant’s cold response: it phosphorylates ICE1, which in turn promotes the ubiquitination and degradation of ICE1 by HOS1 (Figure 2C) [96,97]. In *Arabidopsis thaliana*, phosphorylation by MPK3/ MPK 6 promotes the ubiquitin-dependent degradation of ICE1, whereas in rice, phosphorylation by OsMPK3 enhances ICE1 stability. This species-specific divergence implies evolutionary differentiation in the crosstalk mechanism. However, interspecies comparative analysis of ICE1 phosphorylation residues has not been performed to date.

Trehalose is a stable non-reducing disaccharide. It acts as a glucose storage and transport molecule, an energy source, and a key stress-responsive compound that exerts a protective function under stress conditions. In rice, significantly increasing the expression levels of two key enzymes in the trehalose synthesis pathway—*OsTPS1* (Trehalose-6-Phosphate Synthase 1) and *OsTPP1* (Trehalose-6-Phosphate Phosphatase 1)—can notably raise trehalose content, thereby enhancing the cold tolerance of rice plants [98]. Cold-induced OsPP2C27 (Protein Phosphatase 2C 27) is able to directly dephosphorylate phosphorylated OsMAPK3 and OsbHLH002. This process negatively regulates the OsMAPK3-OsbHLH002-OsTPP1 signaling pathway, preventing the continuous activation of positive regulatory pathways in response to cold stress and maintaining the normal growth of rice plants under low-temperature conditions [99]. Recent studies have revealed that under non-stress conditions, the S-acyltransferase MtPAT9 can interact with MtNAC80 and mediate its S-acylation modification, which in turn facilitates the membrane localization of MtNAC80. Under cold stress conditions, the thioesterase MtAPT1—responsible for deacetylation—oligomerizes into an enzymatically active tetramer. This active MtAPT1 tetramer catalyzes the de-S-acylation of MtNAC80, enabling MtNAC80 to translocate into the nucleus [100]. Once in the nucleus, MtNAC80 directly activates the expression of the *MtGSTU1* gene. This activation promotes the scavenging of malondialdehyde (MDA) and hydrogen peroxide (H_2_O_2_), thereby enabling the plant to resist cold stress [101]. In Chinese cabbage, NO accumulated under cold stress induces the S-nitrosylation of monodehydroascorbate reductase (MDHAR), which significantly enhances the role of brassinosteroid (BR) in the cold tolerance of Chinese cabbage [102].

### 3.3. Drought Stress

When the water potential of the rhizosphere decreases to a certain level, it triggers osmotic stress. This prevents plants from absorbing water from the soil, leading to the massive accumulation of abscisic acid (ABA). The ABA receptors, including pyrabactin resistance (PYR), PYR1-LIKE (PYL), and regulatory components of ABA receptor (RCAR), bind to ABA and interact with class A protein phosphatases (PP2Cs, protein phosphatase 2Cs). This interaction activates class III SNF1-related protein kinase 2 (SnRK2s, sucrose non-fermenting 1-related protein kinase 2s) [103,104,105]. As a type of serine/threonine protein kinase, SnRK2s are widely present in plants. In terms of drought resistance in *Arabidopsis thaliana*, SnRK2.6 (also known as OST1, open stomata 1) regulates the expression of numerous drought-related genes, the activity of ion channels, and metabolic pathways, serving as a hub for plant droughtstress responses. Its activation state directly affects the plant’s ability to cope with drought stress. ABA-activated SnRK2s precisely regulate the ion concentration inside and outside cells through the phosphorylation modification of plasma membrane ion channel proteins [106,107]. This regulation helps maintain cell turgor, controls the opening and closing of stomata, reduces water loss, and optimizes water use efficiency [108,109]. Under the condition of massive ABA accumulation, OST1 directly phosphorylates transcription factors such as ABA-responsive element binding factors (ABF) and ABA-responsive element binding proteins (AREB). This phosphorylation promotes the transcription of a series of drought-resistant genes, which are involved in physiological processes such as maintaining cellular osmotic balance, scavenging reactive oxygen species, and repairing damaged cell membranes, thereby comprehensively enhancing the plant’s viability in arid environments [110,111,112]. Rapidly accelerated fibrosarcoma (RAF)-like kinases play a key role in ABA/drought and osmotic stress signaling pathways by activating the activity of SnRK2s. Under mild drought stress, RAF kinases of the B2 subgroup are activated. They initiate the activation process of SnRK2s through phosphorylation modification. Under more severe drought conditions, dehydration stress-activated RAFs of the B3 subgroup can further enhance the activity of SnRK2s. This indicates that RAF protein kinases of the B2 and B3 subgroups activate ABA-dependent SnRK2s (SnRK2.2/2.3/2.6). In contrast, RAF kinases of the B4 subgroup (RAF16/18/20/24/35/40/42) activate class I SnRK2s (ABA-independent SnRK2s) in an ABA-independent manner. Class I SnRK2s, through directly interacting with, phosphorylate varicose (VCS), a scaffold protein of the mRNA decapping complex. The class I SnRK2s-VCS signaling module plays an important role in regulating the expression of drought-responsive genes as well as root structure and development. After sensing osmotic stress signals, RAF kinases promote the enhancement of SnRK2s activity through phosphorylation. This process can not only proceed independently in the absence of ABA but also synergize with the ABA signaling pathway to jointly regulate the plant’s drought stress response [113,114,115,116,117,118,119]. This dual regulatory mechanism enables plants to cope more flexibly with drought stress with different intensities and durations, thereby improving their viability and adaptability. In addition, studies have found that RAF22 enhances the phosphatase activity of ABI1 through phosphorylation. Conversely, ABI1 can enhance the kinase activity of RAF22 through dephosphorylation, forming a positive feedback loop. Under drought stress conditions, ABA-activated OST1 inhibits the activity of RAF22 through phosphorylation, thereby regulating the balance between plant growth and stress response under drought conditions. The study reveals the dynamic regulatory mechanism among RAF22, ABI1, and OST1 [120]. In terms of transcriptional regulatory modifications, AREB1, AREB2, and ABF3 in ABA-responsive elements (ABRE) are phosphorylated and activated under drought stress, thereby regulating the expression of various drought-responsive genes [112]. In tomatoes, Calcium-dependent protein kinases (CPKs) can perceive and transduce drought signals. By phosphorylating and activating tonoplast sugar transporter 2 (TST2), CPKs promote the accumulation of soluble sugars in cells, thereby enhancing the drought tolerance of tomatoes [121]. OsbZIP23, a member of the ABA signaling pathway, cooperates with histone modification (histone H3K4 trimethylation, H3K4me3) to regulate the expression of drought-responsive genes, thereby positively regulating the drought resistance of rice [122]. Under drought stress, OsWRKY55 can interact with and be activated by four mitogen-activated protein kinases (MAPKs), namely OsMPK7, OsMPK9, OsMPK20-1, and OsMPK20-4, thereby enhancing the drought tolerance of rice at the seedling stage [123]. Drought-activated SnRK2 directly phosphorylates SPCH, a key transcription factor for stomatal development. SnRK2-mediated phosphorylation leads to the degradation of the SPCH protein, thereby inhibiting stomatal development to reduce water loss and improve drought resistance [124]. The transcription factor auxin response factor 7 (ARF7) can induce the asymmetric expression of lateral organ boundaries-domain 16 (LBD16) in lateral root cells, guiding roots to grow toward water. SUMOylation negatively regulates the DNA-binding activity of ARF7. Dry conditions promote the SUMOylation of ARF7, while humid conditions inhibit it, initiating transcriptional regulation and promoting lateral root formation [125]. In rice, deep rooting 1 (DRO1) is negatively regulated by auxin. It participates in the elongation of root tip cells and promotes the downward bending of roots in response to gravity. High expression of *DRO1* directs root growth deeper into the soil, allowing the plant to absorb water from deeper soil layers [126]. Under drought stress, the rice E3 ubiquitin ligase soil-surface rooting 1 (SOR1) can ubiquitinate and degrade indole acetic acid 26 (OsIAA26). Located downstream of the AFB2-auxin-OsIAA9 signaling module, this degradation reduces auxin concentration, increases *DRO1* expression, and enhances drought tolerance [127]. In the latest research on sweet potato, the IbNIEL-IbNAC087 module was found to regulate jasmonic acid (JA)-dependent salt and drought stress responses, in which the RING-type E3 ubiquitin ligase IbNIEL can ubiquitinate and degrades the NAC transcription factor IbNAC087 by ubiquitination, so to reduce JA content and thereby negatively regulating the drought and salt tolerance of sweet potato [128]. In apple, The leucine zipper transcription factor MdHDZ27 can promote the expression of dehydration-responsive genes *MdRD29A* (dehydration 29A) and MdRD29B, while MdBT2, a protein containing BTB-BACK-TAZ domains, can ubiquitinate and degrade MdHDZ27 [129]. Also in apple, the bHLH transcription factor MdbHLH160 can directly upregulate the expression of the dehydration-responsive element-binding factor (DREB) family gene *MdDREB2A-like*, to enhance drought tolerance, whereas MdBT2 can ubiquitinate and degrade MdbHLH160 to negatively regulate drought tolerance (Figure 2D) [130].

### 3.4. Salt Stress

In the plant response to salt–alkali stress, the SOS (Salt Overly Sensitive) signaling pathway is widely regarded as the core module for maintaining ion homeostasis. This pathway mainly consists of four key components: SOS1, SOS2, SOS3, and SCaBP8 (also known as CBL10) [131]. Among them: *SOS1* encodes a plasma membrane-localized Na^+^/H^+^ antiporter, whose function is to effectively extrude excess Na^+^ from the cytoplasm to the extracellular space; SOS2, as a key serine/threonine protein kinase, plays a central role in the signal amplification process; SOS3 and SCaBP8, as calcium sensors belonging to the CBL (calcineurin B-like proteins) family, can sensitively detect changes in cytoplasmic Ca^2+^ concentration induced by salt stress. The classical activation mode of the SOS signal can be summarized as follows: salt stress causes a transient increase in cytoplasmic Ca^2+^; after sensing this change, SOS3 and SCaBP8 bind to SOS2 to form a kinase complex, which relieves the autoinhibitory state of SOS2 and activates its kinase activity; subsequently, the activated SOS2 complex phosphorylates the cytoplasmic tail of SOS1. This process significantly enhances the Na^+^/H^+^ exchange activity of SOS1, thereby accelerating the extrusion of Na^+^ and ensuring the homeostatic balance of cytoplasmic ions (Figure 2A) [131].

Although the classical Ca^2+^-dependent SOS activation mechanism has been widely accepted, recent studies have revealed that under calcium-deficient conditions, SCaBP1 (CBL2) can directly bind to the FISL motif of PKS24 (a member of the SOS2 family) through its C-terminal tail, thereby activating the kinase activity of PKS24. Notably, the phosphorylation of PKS24 at the Thr-211 and Thr-212 sites is crucial for this process [132]. This finding challenges the traditional view that SOS signal activation strictly depends on changes in cytoplasmic Ca^2+^, indicating that plants possess the ability to flexibly activate salt tolerance mechanisms under various ionic environment conditions. Under salt stress, phosphatidylinositol (PI) is converted into phosphatidylinositol 4-phosphate (PI4P). The latter indirectly enhances SOS1 activity by relieving the inhibitory effect on plasma membrane H^+^-ATPase. Meanwhile, phosphatidic acid (PA) can directly bind to SOS2, thereby promoting the localization of SOS2 on the plasma membrane and its stable activation. These studies reveal the tight coupling between phospholipid metabolism and the SOS pathway, highlighting the key regulatory role of dynamic changes in the phospholipid environment in salt stress signal transduction [133].Phosphorylation of SCaBP family members is a conserved regulatory mechanism. PKS24-mediated phosphorylation of SCaBP1 at the Ser-216 site enhances the binding affinity between SCaBP1 and PKS24 and stabilizes the formation of the signal complex [134]. Under non-stress conditions, 14-3-3 proteins can bind to SOS2, thereby inhibiting its kinase activity and effectively blocking the downstream activation process of SOS1 [135]. When salt stress occurs, the binding between 14-3 and 3 proteins and SOS2 decreases, allowing the activation cascade to be initiated. Two protein phosphatases, namely PP2C.D6 and PP2C.D7, have been further identified. Under normal conditions, these two phosphatases inhibit the activity of SOS1 through dephosphorylation [136]. However, under salt stress conditions, SCaBP8 can inhibit the activity of these two phosphatases, relieving their negative effect on SOS1 and enhancing the ability of Na^+^ efflux.

In the plasma membrane Na^+^/H^+^ antiport system, the phosphorylation regulatory mechanism of SOS1 cytoplasmic tail determines the antiport activity under salt stress, and this process is activated and regulated by the SOS2-SOS3 complex [137]. Structural biology studies have resolved the cytoplasmic domain (CPD) of SOS1 at high resolution, revealing that it contains three important structural regions: the inhibitory functional domain (IFD) with an inhibitory role, the cyclic nucleotide binding domain-like (CNBD-like) domain with an activating role, and the autoinhibitory domain (AI) with an autoinhibitory function. Among these regions, the IFD cooperates with the AI domain to maintain SOS1 in a low-activity state. After salt stress triggers the phosphorylation by SOS2, the AI domain is released, which promotes the conformational change in SOS1 and increases the Na^+^/H^+^ exchange rate [138]. In the regulation of phospholipid metabolism and plasma membrane H^+^-ATPase, phosphatidic acid (PA) acts as a salt stress signaling molecule that can directly bind to SOS2, promoting the activation of SOS2 and its recruitment to the plasma membrane region. In this process, PA not only functions as a signaling mediator but also can bind to and activate MAPK6. The activated MAPK6 is capable of phosphorylating SOS1, thereby promoting Na^+^ efflux and enhancing the salt tolerance of plants [133,139].

In terms of ubiquitination, three genes encoding E2 (AtUBC7, AtUBC13, and AtUBC14) have been shown to respond to high-salt stress and may play a role in the degradation process of misfolded proteins in the endoplasmic reticulum [140].

Overexpression of the RING-type E3 gene *IbATL38* from sweet potato (*Ipomoea batatas*) in *Arabidopsis thaliana* enhances the latter’s salt tolerance. It upregulates the expression of stress-responsive genes in anABA-signaling-dependent manner and reduces the accumulation of reactive oxygen species (ROS) [141]. In *Arabidopsis thaliana*, the RING-type E3 genes *AtATL31* and *AtATL6* negatively regulate plant salt tolerance, but this regulation is independent of the ABA signaling pathway [142]. In rice (*Oryza sativa*), the RING-type E3 gene *OsSIRP4* is highly expressed under salt stress. In transgenic rice overexpressing *OsSIRP4*, the activity of enzymes related to ROS scavenging is decreased, the accumulation of proline and soluble sugars is significantly reduced, and the expression of genes related to K^+^/Na^+^ homeostasis is downregulated. Additionally, OsSIRP4 can interact with the peroxisome biogenesis factor OsPEX11, thereby negatively regulating the salt stress response [143]. Under salt stress, the HMG box-containing transcription factor MdHMGB15 in apple enhances the expression of *MdXERICO*, encoding a RING-type E3 ubiquitin ligase, thereby mediating the ubiquitination and degradation of MdNRP and improving salt tolerance [144]. In soybean, an E3 ubiquitin ligase, GmCHYR16, negatively regulates salt tolerance by mediating the ubiquitination and degradation of GmERF71, an AP2/ERF transcription factor [145]. A study on *Arabidopsis thaliana* has shown that the ubiquitin-conjugating enzyme UBC34 can ubiquitinate proton-pumping pyrophosphatase (AVP1), a key stress-responsive factor, thereby reducing its activity and negatively regulating plant salt tolerance [146].

In recent years, S-nitrosylation has been uncovered as a key regulatory mechanism in plant salt tolerance. In tomato, salt stress induces the accumulation of NO in roots. High concentrations of NO lead to the S-nitrosylation of γ-aminobutyric acid transaminase 1 (SlGABA-TP1) at cysteine residues 316/258/316 (Cys316/258/316), which reduces its enzymatic activity and increases γ-aminobutyric acid (GABA) content. Through interaction with SlALMT14, GABA decreases the efflux of malic acid from roots, thereby affecting the plant’s saline–alkali tolerance [147]. NO stimulates the transcription of caffeic acid O-methyltransferase (COMT) and the biosynthesis of melatonin, which scavenges excess NO at the proteomic level and alleviates nitrosative damage. Under saline–alkali stress, plasma membrane-localized H^+^-ATPase 2 (HA2) undergoes S-nitrosylation at cysteine-206 (Cys206), impairing its interaction with 14-3-3 protein 1 (TFT1). This S-nitrosylation of HA2 leads to reduced enzyme activity and decreased H^+^ efflux, thereby compromising the plant’s saline–alkali tolerance. Conversely, melatonin produced by COMT mitigates the S-nitrosylation of HA2, restoring its function and the saline–alkali tolerance of tomato plants [148]. Also in tomato, drought and salt stresses induce the production of NO, which activates the expression of genes encoding Δ1-pyrroline-5-carboxylate synthetase (SlP5CS) and Δ1-pyrroline-5-carboxylate reductase (SlP5CR), thereby promoting proline accumulation to cope with stresses. The S-nitrosylation of SlP5CR enables tomato plants to better adapt to changes in NAD(P)H levels under stress conditions, enhancing SlP5CR activity and proline biosynthesis during stress exposure [149]. NO-mediated S-nitrosylation of BRI1-associated kinase 1 (BAK1) enhances its interaction with BRASSINOSTEROID INSENSITIVE 1 (BRI1), promoting the activation of the brassinosteroid (BR) signaling pathway and thereby improving the salt tolerance of tomato plants [150].

In terms of thePTM-mediated regulation of NAC transcription factors, tomato can mediate the ubiquitination and degradation of SlNAC35, a member of the NAC transcription factor family, via the E3 ubiquitin ligase SlMIEL1. This process enhances the transcription of *allene oxide cyclase* (*AOC*) gene, leading to jasmonic acid (JA) accumulation and thereby improving salt tolerance [151]. In apple, the NAC transcription factor MdNAC104 can be ubiquitinated and degraded via the 26S proteasome pathway, which increases the γ-aminobutyric acid (GABA) content in plant roots and thereby enhances salt tolerance [152]. In soybean, cells can directly perceive and transduce reactive oxygen species (ROS) signals through hydrogen peroxide (H_2_O_2_)-mediated post-translational modifications (PTMs) of cysteine residues in proteins. The cysteine residues of NAC WITH TRANS-MEMBRANE MOTIF1-LIKE 1 (GmNTL1) are oxidatively modified by H_2_O_2_, facilitating its release from the endoplasmic reticulum (ER) membrane and translocation to the nucleus. By binding to and activating the promoter of the *respiratory burst oxidase homolog B* (*GmRbohB*) gene, GmNTL1 initiates the amplification of downstream ROS production, forming a positive feedback loop that precisely regulates its own activity. Salt stress-induced oxidation of GmNTL1, via H_2_O_2_-mediated post-translational oxidative modification, promotes its nuclear localization and transcriptional activity, thereby enhancing the salt tolerance of soybean [153].

### 3.5. PTMs in ABA Signaling Transduction

**Figure 3:**
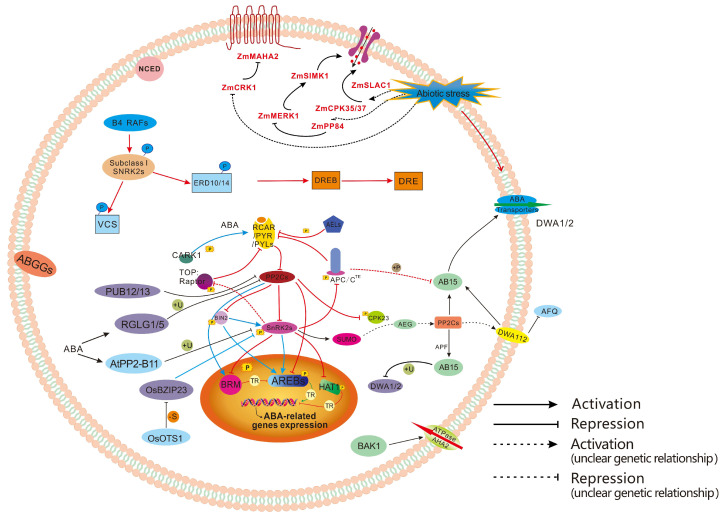
PTMs in ABA signaling transduction.

As one of the classic plant hormones, ABA is not only involved in plant growth and development processes, such as seed dormancy and germination, root system development, leaf senescence, and floral transition, but also plays a crucial role in plant stress responses [154,155]. Moreover, PTMs of proteins exert a significant function in the ABA signal transduction pathway. In the absence of ABA or stress signals, the key component of energy metabolism, the TOR (Target of Rapamycin) kinase complex, phosphorylates PYL1/4, thereby blocking the ABA signal. When plants are subjected to stress, SnRK2s are activated and phosphorylate Raptor B, the regulatory subunit of TOR. This leads to the inhibition of TOR kinase activity, preventing it from activating energy regulation, which in turn inhibits plant growth and promotes plant survival under stress conditions. These results indicate that the TOR kinase complex and the ABA signal exert antagonistic effects to regulate plant growth and survival under stress [156,157]. *Arabidopsis* casein kinases AELs (Arabidopsis EL1-like proteins, AEL1-AEL4) phosphorylate Ser136 of PYL1 and Ser109 of PYR1. The deletion of AELs reduces the ubiquitination of PYL1/PYR1, resulting in slower degradation of PYR/PYLs. This suggests that there exists a phosphorylation-mediated ubiquitination degradation pathway for PYL1/PYR1. Interestingly, phosphorylation at different sites of the receptor may exert opposite effects [158]. CARK1 (cytosolic ABA receptor kinase 1) phosphorylates the threonine residues at positions 77/78 (T77/T78) of PYL8/PYR1, which enhances the stability of PYL8/PYR1 and strengthens its inhibitory effect on PP2Cs, thereby promoting ABA signal transduction [159]. Phosphorylation of PYL1 at Ser136 by AEL kinases promotes its ubiquitination and degradation, whereas phosphorylation of PYL8 at T77/T78 by CARK1 enhances its stability. Such contradictions may stem from the functional specificity of phosphorylation residues—Ser136 is located in the ABA-binding domain of PYL1, and its phosphorylation may impair ABA-binding capacity and recruit E3 ubiquitin ligases; in contrast, T77/T78 reside in the dimerization domain of PYL8, and their phosphorylation may strengthen the interaction between PYL8 and PP2Cs, thereby inhibiting its degradation. However, systematic identification of phosphorylation residues across different members of the PYL family is currently lacking (Figure 3). Moreover, whether phosphorylation of PYLs by different kinases exhibits a competitive relationship, and whether ABA modulates the crosstalk balance by affecting kinase activity, remain insufficiently investigated. In addition, whether phosphorylation modification of PYL receptors displays species-specific divergence, and whether their regulatory patterns in crops are consistent with those in Arabidopsis thaliana, still require more empirical data to support.

When plants perceive the ABA signal, the inhibition of SnRK2s is relieved. SnRK2s recover partial activity through intramolecular interactions between their kinase catalytic domain and regulatory domain, and then autophosphorylate multiple amino acid residues in the activation loop to restore full kinase activity, thereby phosphorylating downstream substrates [160].In addition to autophosphorylation of SnRK2s, multiple kinases such as BIN2 (brassinosteroid insensitive 2), BAK1 (BRI1-associated receptor kinase 1), HT1 (high leaf temperature 1), and casein kinase CK2 (casein kinase 2) can also phosphorylate SnRK2s. BIN2 belongs to the GSK3 (glycogen synthase kinase 3) kinase family and regulates a variety of signaling processes, including inhibiting BR signal transduction, suppressing auxin signal transduction, and modulating stomatal movement [161,162,163,164]. It is one of the key components in the BR signaling pathway [164]. In the ABA signaling pathway, BIN2 phosphorylates and activates SnRK2.2/2.3, and at the same time, it also phosphorylates and activates ABI5 [165]. Latest studies have shown that ABI1/2 (ABA-insensitive 1/2), two key PP2Cs in the ABA pathway, inhibit BIN2 kinase activity by dephosphorylating BIN2. These results indicate that BIN2 is a key factor integrating the two major hormone signaling pathways of BR and ABA [166].BAK1, another kinase in the BR signaling pathway, also phosphorylates SnRK2.6/OST1 (open stomata 1) and antagonizes ABI1 in regulating the phosphorylation of OST1 and stomatal movement [161]. CK2 is a highly conserved protein kinase that forms heterodimers or tetramers composed of catalytic subunits and regulatory subunits [167]. ZmCK2 from maize phosphorylates amino acid residues in the C-terminal domain of ZmOST1, promoting the binding of ZmOST1 to PP2Cs, thereby inhibiting the activity of OST1 and ABA signal transduction [162]. HT1 is a negative regulator of CO_2_-induced stomatal closure; it inhibits the kinase activity of OST1 through phosphorylation, thereby suppressing stomatal closure [163]. Furthermore, kinases such as MPKs and phosphatases such as PP2A and PP1 all affect the phosphorylation level and kinase activity of SnRK2s, but whether they directly regulate the phosphorylation of SnRK2s requires further verification (Figure 3) [168]. The traditional view holds that the activation of SnRK2s relies on autophosphorylation following the relief of PP2C-mediated inhibition, whereas recent studies have revealed that kinases such as BIN2, BAK1 and BIK1 can directly phosphorylate SnRK2s and activate their activity [169]. The hierarchical relationship and synergistic mechanism between these two activation modes remain unclear—does the relief of PP2C inhibition provide a prerequisite for kinase-mediated phosphorylation in the ABA signaling pathway? Or do these two pathways function independently and target distinct SnRK2 members? In addition, SnRK2s harbor multiple phosphorylation sites; whether different kinases modify distinct sites and elicit divergent activity effects has not been elucidated. The core of this controversy lies in the insufficient understanding of the hierarchical regulatory network underlying SnRK2 activation. Future research should employ in vitro reconstitution assays and residue-specific phosphorylation antibodies to clarify the sequential order and functional contribution of different phosphorylation events.

AREBs/ABFs belong to the basic leucine zipper (bZIP) family of transcription factors, such as ABI5, ABF1-ABF4, OsbZIP23, and OsbZIP46 [111]. They activate or repress the expression of their target genes by binding to the ABA response element (ABRE). AREBs/ABFs are phosphorylated by SnRK2s and directly dephosphorylated by ABI1/2 [170]. Most transcription factors have their transcriptional activity activated after being phosphorylated by SnRK2s. For instance, in rice, OsbZIP23 is phosphorylated by SAPK2 (a homolog of SnRK2s in rice that participates in ABA signal transduction), which activates its transcriptional activity. This activation then promotes the expression of *OsNCED4* and the synthesis of ABA [171]. Recent studies have shown that after ABFs are phosphorylated by SnRK2s, they promote the expression of *ABI1/2*. Subsequently, the protein levels of ABI1/2 increase, enhancing the dephosphorylation of ABFs and thereby repressing the transcriptional activity of ABFs [172]. This forms a feedback pathway that precisely regulates the ABA signal and mediates the desensitization response to ABA. Some transcription factors, however, have their transcriptional activity inhibited after phosphorylation by SnRK2s. Under normal conditions, for example, unphosphorylated HAT1 (homeodomain-leucine zipper protein 1) binds to the promoter of *NCED3*, repressing the expression of *NCED3* and the synthesis of ABA. Under drought conditions, HAT1 is phosphorylated by SnRK2s, which inhibits its transcriptional repressor activity. This leads to an increase in ABA content in plants, enabling them to respond to drought stress [173].In addition to SnRK2s and PP2Cs, a variety of other kinases or phosphatases are also involved in regulating the phosphorylation modification of AREBs/ABFs. In pepper, CaSnRK2.4 can activate the NAC transcription factor CaNAC035 through phosphorylation to upregulate two key ABA biosynthesis genes (CaAAO3 and CaNCED3), thereby enhancing cold stress tolerance [174].

During the process of ABA promoted stomatal closure, SnRK2s phosphorylate and activate SLAC1, thereby facilitating anion (A^−^) efflux; ABI1 directly dephosphorylates SLAC1, inhibiting stomatal closure. Meanwhile, SnRK2s phosphorylate and inhibit the activity of KAT1, preventing potassium ion (K^+^) influx [175]. SnRK2s also phosphorylate the bHLH-type transcription factor AKS1 (ABA-responsive kinase substrate 1), inducing AKS1 to depolymerize into a monomeric form. This depolymerization causes AKS1 to lose the ability to bind to its target gene *KAT1*, thereby inhibiting the expression of *KAT1* [176]. Beyond this pathway, ABI1 also inhibits the phosphorylation of SLAC1 by CPK23 (calcium-dependent protein kinase 23) through dephosphorylating CPK23, forming a SnRK2s-independent pathway that regulates stomatal movement; CPK6 also phosphorylates SLAC1, partially substituting for the function of OST1 [177]. The kinase GHR1 (guard cell hydrogen peroxide-resistant 1) phosphorylates and activates SLAC1, participating in ABA-regulated stomatal closure. This process is inhibited by ABI2 but not by ABI1 [178]. Additionally, during CO_2_-induced stomatal closure, HT1 not only inhibits OST1 but also suppresses GHR1 and SLAC1 through phosphorylation. In maize, ZmCPK35 and ZmCPK37 can phosphorylate ZmSLAC1 to regulate guard cell channel activity [179]. CALCIUM-DEPENDENT PROTEIN KINASE RELATED KINASE 1 (ZmCRK1) interacts with the plasma membrane proton ATPase ZmMHA2, and inhibits its proton pump activity by phosphorylating the Ser-901 site of ZmMHA2, leading to impaired ABA-induced H^+^ efflux and negatively regulating drought tolerance [180]. In contrast, CO_2_ can promote the phosphorylation of MPK4/MPK12, which in turn inhibits the kinase activity of HT1 [181]. SnRK2s also phosphorylate other functional proteins, enabling plants to exhibit a variety of physiological changes after perceiving the ABA signal (Figure 3).

ABA receptors PYR1/PYL/RCAR, which are specific recognition receptors, along with their downstream PP2Cs and SnRK2s, are all core components of the ABA signaling pathway and subjected to PTMs. Specifically, the RING-type E3 ubiquitin ligase RSL1 (ring finger of seed longevity 1) can directly interact with PYR1 and PYL4 to mediate their ubiquitination and degradation, thereby reducing plant sensitivity to ABA [182]. The ubiquitin E2-like protein VPS23A (vacuolar protein sorting 23A) can recognize the ABA receptor complex RSL1-PYL4 and its K63-linked ubiquitin chain, promoting the complex to enter the vesicular transport pathway and thus affecting its subcellular localization and stability [183]. In *Arabidopsis thaliana*, four E3 ubiquitin ligases—SINAT1 (seven in absentia of *Arabidopsis thaliana* 1), SINAT2, SINAT3, and SINAT4—can regulate the ubiquitination and proteasomal degradation of VPS23A and FREE1, thereby reducing the stability of these two proteins and positively regulating the ABA signaling pathway [184]. Meanwhile, the RING-type E3 ubiquitin ligase XBAT35 (E3 ligase XB3 ortholog 5 in *Arabidopsis thaliana*) can also catalyze the ubiquitination of VPS23A, which is subsequently degraded by the proteasome, further exerting a positive regulatory effect on the ABA signaling pathway [185]. Two deubiquitinating enzymes involved in deubiquitination—UBP12 (ubiquitin-specific protease 12) and UBP13—enhance the stability of VPS23A by catalyzing its deubiquitination [186]. This increased stability of VPS23A in turn promotes the degradation of ABA receptors, thereby negatively regulating the ABA signaling pathway. Additionally, UBP12 and UBP13 can catalyze the deubiquitination of XBAT35, leading to the stabilization of XBAT35 protein. Notably, XBAT35 competes with VPS23A for binding to UBP12 and UBP13, which enables the fine-tuning of VPS23A ubiquitination and degradation [187]. The E2 ubiquitin-conjugating enzyme UBC26 can directly bind to PYL4 and regulate the ubiquitination and degradation of PYL4, thereby negatively regulating the ABA signaling pathway. Importantly, this regulatory process requires the joint participation of the RING-type E3 ubiquitin ligases RFA1 (RING finger ABA-related 1) and RFA4 [188].

Ubiquitination of the protein phosphatase family (PP2C) is involved in the signal transduction pathway of ABA. U-box-type E3 ligases (PUBs, plant U-box E3 ligases) are involved in the ubiquitination and degradation of ABI1 [189]. RGLG1 (RING domain ligase 1) and RGLG5 from the RING-type E3 ubiquitin ligase family participate in the ubiquitination of three PP2C proteins, namely AHG3, ABI2, and HAB2 [190]. Additionally, the BPM (BTB/POZ and MATH domain proteins) subunits (BPM3 and BPM5) of the E3 ubiquitin ligase CRL3s (Cullin3-RING-based E3 ligases) can also interact with PP2C protein phosphatases such as ABI1, ABI2, and HAB1 to regulate their ubiquitination [191].

SnRK2, a key kinase in the ABA signaling pathway, is also regulated by ubiquitination. AtPP2-B11 is an F-box protein in the SCF (SKP1/Cullin/F-box) E3 ubiquitin ligase complex; it can ubiquitinate SnRK2.3 and promote its degradation by the 26S proteasome [192]. HOS15 (high osmotic stress 15), a substrate receptor protein in the CUL4-DDB1 E3 ubiquitin ligase complex, can facilitate the ubiquitination of SnRK2.6/OST1, which is then degraded by the 26S proteasome [193].

The stability of SnRK2.6/OST1 is also regulated by the de-SUMOylation mediated by the SUMO protease ESD4 and its interacting protein NUA (nuclear pore anchor), thereby negatively regulating the ABA signaling pathway [194,195]. Therefore, we hypothesize that the reason why SUMOylation enhances protein stability is most likely by competing with ubiquitin for binding to the lysine residues of proteins.

Transcriptional regulators in the ABA signaling pathway are also involved in the regulation of ubiquitination. Studies have shown that the transcription factor ABI3 (abscisic acid-insensitive 3) with a B3 domain can bind to the E3 ubiquitin ligase AIP2 (ABI3-interacting protein 2) and the U-box-type E3 ubiquitin ligase PUB9 (plant U-box 9), and is subsequently degraded by the proteasome, thereby negatively regulating the ABA signaling pathway [196,197,198]. The lysine at position 344 in the C3 domain of the bZIP transcription factor ABI5 (abscisic acid-insensitive 5) is ubiquitinated by the RING-type E3 ubiquitin ligase KEG; this ubiquitination leads to ABI5 degradation by the proteasome, which negatively regulates the ABA signaling pathway [199,200]. Additionally, the RING-type E3 ubiquitin ligase SDIR1can ubiquitinate SDIRIP1 (SDIR1-interacting protein 1), resulting in SDIRIP1 degradation by the 26S proteasome. This process further upregulates the transcriptional level of the *ABI5* gene, thereby positively regulating the ABA signaling pathway [201,202]. The lysine at position 391 of ABI5 can also be SUMOylated under the catalysis of the SUMO ligase SIZ1. This SUMO modification protects ABI5 from proteasomal degradation, thus negatively regulating the ABA signaling pathway [203]. MYB30 is a negative regulator of the ABA signaling pathway. The lysines at positions 165 and 283 of MYB30 can be ubiquitinated and degraded by the RING-type E3 ubiquitin ligase RHA2b (RING-H2 finger protein A2b); MYB30 can also be ubiquitinated by the E3 ligase MIEL1 (MYB30-interacting E3 ligase 1) [204,205]. Moreover, SIZ1 can catalyze the SUMOylation of the lysine at position 283 of MYB30. Similar to its modification of ABI5, this SUMOylation also enhances the stability of MYB30 [206].

## 4. PTM Crosstalk

In cells, crosstalk occurs between any two or more different PTMs through two molecular modes: (1) Different modifications coordinately regulate the molecular function of the same substrate protein. In this mode, one type PTM affects other PTM(s) at the same amino acid site(s) or adjacent amino acids; (2) Multiple enzymes with “writer” or “eraser” functions sequentially use each other as substrates to alter enzyme activity by adding or removing modifications, and to form positive regulatory or feedback regulatory loops to exert their functions [207,208,209].

### 4.1. Crosstalk Between Phosphorylation and Ubiquitination

For some proteins, after being modified by phosphorylation, they become the targets of ubiquitination to induce the degradation of substrate proteins through cis-regulation, a process termed “phosphodegron (a phosphorylated motif that mediates ubiquitination-dependent degradation)” [210]. This process usually involves a specific phospho-motif on the substrate protein serving as an anchoring site for the ubiquitin ligase. When the motif is phosphorylated, it induces the binding of the ubiquitin ligase to the substrate and initiates the ubiquitination-mediated degradation process. For example, when *Arabidopsis thaliana* is subjected to high-concentration metal ion stress, its iron-regulated transporter 1 (IRT1) is regulated by activating the phosphodegron mechanism [211]. After IRT1 is modified by phosphorylation, it provides a binding region for the E3 ubiquitin ligase IRT1-degradation factor 1 (IDF1). IDF1 ubiquitinates two lysine sites of IRT1, thereby inducing the endocytosis and degradation of IRT1. Under high-carbon and low-nitrogen nutrient stress, at least one amino acid residue of ATL31 in *Arabidopsis thaliana* is phosphorylated by CIPK [212]. After 14-3-3 protein binds to the E3 ligase ATL31, it induces the sequential phosphorylation of 4 amino acid sites at the C-terminus of ATL31 [213]. Moreover, the interaction between these two proteins is crucial for the ubiquitination of ATL31 and the degradation of 14-3-3 protein induced by ATL31 [214]. This indicates that the phosphorylation of E3 ubiquitin ligase can activate its interaction with substrates.

Most extracellular signals are transmitted into the cell through membrane-localized receptor kinases. Generally, once receptor kinases are activated by ligands, they undergo phosphorylation and ubiquitination modifications, which in turn induce the intracellular translocation, turnover, or degradation of receptor kinases and facilitate signal transmission. Studies have shown that the signaling pathways of the flagellin-sensitive 2 (FLS2) receptor and the brassinosteroid insensitive 1 (BRI1) receptor are both regulated by the “phosphorylation–ubiquitination modification” crosstalk mode [215,216]. When these two receptor kinases sense their respective ligands (flagellin and BR, respectively), FLS2 and BRI1 form complexes with BAK1 kinase, respectively, undergo endocytosis into the cell, and jointly activate downstream pathways. Importantly, both flagellin and BR signals phosphorylate the conserved amino acid residues of the E3 ubiquitin ligases PUB12/13, thereby activating the interaction between PUB12/13 and their respective receptors (FLS2 and BRI1) and ultimately leading to their degradation. Although the mechanisms by which FLS2 and BRI1 recruit ubiquitin ligases are different, phosphorylation of FLS2 and BRI1 plays a decisive role in the PUB12/13-mediated degradation pathway [215,216]. In the ABA signaling pathway, when the protein kinase SnRK2.3 is activated and phosphorylates the substrate protein PP2-B11, it induces the proteasomal degradation of PP2-B11 through a ubiquitination-dependent pathway, thereby regulating the ABA signaling pathway and abiotic stress response [31].

### 4.2. Crosstalk Between Phosphorylation and SUMOylation

The first reported protein with the “phosphorylation–SUMOylation “ crosstalk regulatory mode is CESTA, a core transcription factor in the brassinosteroid signaling pathway [217]. Studies have found that treating plants with BR leads to the SUMO1 and SUMO1-like ubiquitin modification of lysine 72 (K72) in CESTA, which in turn induces the nuclear translocation of CESTA and activates its transcriptional activity. However, CPK can attenuate this ubiquitin-like modification process and nuclear translocation effect by phosphorylating CESTA [217]. There are a few reports on this type of crosstalk in plant immunity, but no such protein with a role in abiotic stress response has been reported.

### 4.3. Crosstalk Between Ubiquitination and SUMOylation

Ubiquitination and ubiquitin-like can either antagonize or synergistically regulate each other [218]. Although both modifications involve the E1-E2-E3 signaling pathway, their molecular regulatory modes are different. Furthermore, ubiquitination and SUMOylation compete for the same lysine site. Early studies found that under salt stress, SUMOylation of lysine 65 (K65) in the DELLA protein repressor of GA (RGA) promotes the expression of this RGA [219]. When this site is mutated to arginine, the expression level of RGA remains unchanged. Further studies have shown that the lysine site K65 of RGA is co-regulated by two modifications, namely SUMOylation and ubiquitination, and SUMOylation can protect RGA from gibberellin-mediated degradation via the ubiquitination pathway. Another study found that SUMOylation and ubiquitination compete for 4 lysine sites (Lys50, Lys276, Lys281, and Lys291) of the protein kinase RACK1B, and the SUMOylation of these 4 lysine sites enhances the sensitivity of RACK1B to ABA-mediated ubiquitination and degradation [220].

### 4.4. Crosstalk Between Phosphorylation, Ubiquitination, and SUMOylation

The synergistic regulatory mode of three PTMs (phosphorylation, ubiquitination, and SUMOylation) was first reported in animal cells. These three modifications synergistically regulate the genotoxic stress response mechanism and induce processes such as the nuclear localization of the kinase subunit of the NEMO protein [221]. A similar regulatory mechanism also exists in plants. SnRK1 kinase can form a complex with SnRK2 and PP2C, participating in the ABA-dependent environmental response pathway [222]. The activation of SnRK1 depends on the threonine phosphorylation in the T-loop region. The phosphorylation of this site induces the E3 ubiquitin-like ligase SIZ1 to act on multiple subunits of SnRK1 through the ubiquitin-like modification process, leading to the degradation of SnRK1 [223]. This study indicates that SnRK1 exerts its function through SUMOylation induced by the phosphorylation process and the subsequent degradation pathway, and weakens the further transmission of adverse signals to downstream components through a negative feedback regulation mode.

## 5. Conclusions and Perspectives

PTMs of proteins can regulate protein structure, dynamics, and biological functions. They serve as an important regulatory mechanism for plants to rapidly respond to environmental stimuli during their life activities. Given that PTMs can quickly and accurately activate, perceive, integrate, and transmit extracellular signals to downstream pathways, genes encoding proteins subjected to PTMs are ideal targets of genetic engineering for breeding stress-resilient crops.

Regulating gene expression in plants can be effectively achieved using tools such as CRISPR-mediated gene editing and RNA interference (RNAi) [224]. For protein-coding genes, their function can be modulated at multiple levels: DNA, mRNA, and protein. CRISPR-mediated gene editing technologies are powerful tools for modifying crop DNA [225], altering protein sequences, and modifying epigenetic marks [226,227], thereby influencing gene expression level, protein abundance, translation efficiency, and protein activity. For the genes subject to PTMs, at the transcription level, their expression level can be altered by targeting the cis-regulatory elements of promoters; at the post-transcriptional level, their RNA level can be modulated by RNAi or miRNA mimics; at the post-translational level, the abundance and/or function of the protein can be manipulated by targeting the PTM sites [228]. Together, these approaches enable quantitative and qualitative control of protein synthesis.

Beyond protein synthesis, plants also regulate protein homeostasis at multiple levels (including trafficking, localization, and degradation) through PTMs. Protein condensation mediated by phase separation and phase transition is a key process in protein homeostasis regulation [229,230,231,232], and this process plays important roles in plant development and responses to environmental stresses [233,234]. The homeostasis of all proteins, including condensation-prone proteins, is primarily regulated by protein degradation mechanisms, mainly the ubiquitin–proteasome system (UPS) and the autophagy system [235,236]. The UPS mediates the transfer of ubiquitin to substrates through the coordinated action of E1 activating enzymes, E2 conjugating enzymes, and E3 ligases, which typically leads to substrate polyubiquitination and subsequent degradation by the proteasome [226,231,232]. In contrast, the autophagy system is responsible for degrading not only proteins but also organelles to maintain cellular homeostasis [237]. A recent study developed the Targeted Condensation-prone-protein Degradation (TCD) strategy using the E3 ubiquitin ligase E3TCD1. The X-E3TCD1 fusion protein acts as a genetically engineered degrader that can selectively target endogenous proteins with condensation propensity [238]. Proteolysis-targeting chimeras (PROTACs) are defined as heterobifunctional constructs comprising two distinct ligands: one that specifically engages a protein of interest (POI) and another capable of recruiting an E3 ubiquitin ligase. Through chemical induction of proximity between the POI and the recruited E3 ligase, the POI undergoes ubiquitination, followed by its selective degradation via the ubiquitin–proteasome system (UPS) [239]. The development of PROTACs for crop improvement via E3 ubiquitin ligases remains in its nascent stage. Notably, the homologous substrate recognition subunits of E3 ubiquitin ligases serve as promising candidates for integration into PROTAC design frameworks. By mediating the ubiquitination of diverse protein substrates, these subunits modulate a broad spectrum of cellular processes in plants, thereby establishing themselves as valuable resources for advancing PROTAC technologies. Under stress conditions, the abundance and composition of E3 ubiquitin ligases exhibit distinct variations across different plant organs and crop cultivars. Omics-based approaches—including transcriptomics and proteomics—are indispensable for characterizing the expression profiles of E3 ubiquitin ligases under specific contexts. Such investigations would lay the foundation for customizing PROTAC systems to suit targeted scenarios, such as different crop cultivars or specific plant organs. Nevertheless, future development efforts must adequately address the inherent diversity of E3 ubiquitin ligases while further elucidating the functional mechanisms and structural features of plant-specific E3 ubiquitin ligases [240].

Despite targeting E3 ubiquitin ligases, key regulators of environmental signal responses, as the key molecular targets and technical scaffolds, will significantly advance the understanding and manipulation of abiotic stress responses in crops. In future research, it is essential to integrate multi-omics technologies to identify more PTM targets associated with strong stress tolerance and high yield. Meanwhile, combining TCD/PROTACs with CRISPR-mediated gene editing to precisely modify proteins involved in PTMs holds high promise for crop improvements, but the development of artificial degradation systems based on the two technologies still faces numerous challenges in terms of crop genetic improvement.

## Data Availability

No new data were generated in association with this article.
